# Indoor- and Outdoor-Generated Particles: Koenig et al. Respond

**Published:** 2005-09

**Authors:** Jane Koenig, Ryan Allen, Tim Larson, Sally Liu

**Affiliations:** University of Washington Seattle, Washington, E-mail: jkoenig@u.washington.edu

We appreciate Moshammer’s comments and his interest in our research. We have several points to raise in reply.

In our article (Koenig et al. 2005), we stated that indoor sources are known to affect airway inflammation. We recognized that indoor sources vary greatly and that 19 homes may not provide a sufficient sample size to allow for a robust association. It is true that the children in our study spent substantial time away from home. We now have additional data from a panel of 16 adults (average age of 75 years) who did not commute or leave home regularly; in these adults we found the same coefficient with eNO (exhaled nitric oxide) versus outdoor PM_2.5_ (particulate matter ≤2.5 μm in aerodynamic diameter) as in the research in question (Jansen et al. 2004). In addition, Ebelt et al. (2005) found lung function decrements only with ambient particles in a group of non-smoking 54- to 86-year-old adults. These results provide additional evidence of an ambient-only pulmonary effect among individuals who spent relatively little time away from home.

Regarding smoking status, one inclusionary criterion for our study was to be a non-smoker and live with nonsmokers; thus smoking is not an important indoor source of particles in these residences. Children in the Seattle school district do not go home for lunch. However, it is true that our exhaled breath samples were taken 1–2 hr after the commute home (Liu et al. 2003). On average, the time between morning commute and eNO collection was 9 hr; between afternoon commute and breath collection was about 2 hr. We are now looking at the short-term lag structure. Using a polynomial distributed lag model, we found that PM_2.5_ was associated with the eNO for up to 10–12 hr before the eNO measurement (Mar et al., in press).
